# A Simulation Model for Designing Effective Interventions in Early Childhood Caries

**DOI:** 10.5888/pcd9.110219

**Published:** 2012-03-01

**Authors:** Gary B. Hirsch, Burton L. Edelstein, Marcy Frosh, Theresa Anselmo

**Affiliations:** Learning Environments, Wayland, Massachusetts; Columbia University College of Dental Medicine; Dr Edelstein is also president of the Children's Dental Health Project; Children’s Dental Health Project, Washington, DC; San Luis Obispo County Health Agency, San Luis Obispo, California. At the time of this study, Ms Anselmo was affiliated with the Colorado Department of Public Health and Environment, Denver, Colorado

## Abstract

**Introduction:**

Early childhood caries (ECC) — tooth decay among children younger than 6 years — is prevalent and consequential, affecting nearly half of US 5-year-olds, despite being highly preventable. Various interventions have been explored to limit caries activity leading to cavities, but little is known about the long-term effects and costs of these interventions. We developed a system dynamics model to determine which interventions, singly and in combination, could have the greatest effect in reducing caries experience and cost in a population of children aged birth to 5 years.

**Methods:**

System dynamics is a computer simulation technique useful to policy makers in choosing the most appropriate interventions for their populations. This study of Colorado preschool children models 6 categories of ECC intervention — applying fluorides, limiting cariogenic bacterial transmission from mothers to children, using xylitol directly with children, clinical treatment, motivational interviewing, and combinations of these — to compare their relative effect and cost.

**Results:**

The model projects 10-year intervention costs ranging from $6 million to $245 million and relative reductions in cavity prevalence ranging from none to 79.1% from the baseline. Interventions targeting the youngest children take 2 to 4 years longer to affect the entire population of preschool-age children but ultimately exert a greater benefit in reducing ECC; interventions targeting the highest-risk children provide the greatest return on investment, and combined interventions that target ECC at several stages of its natural history have the greatest potential for cavity reduction. Some interventions save more in dental repair than their cost; all produce substantial reductions in repair cost.

**Conclusion:**

By using data relevant to any geographic area, this system model can provide policy makers with information to maximize the return on public health and clinical care investments.

## Introduction

Early childhood caries (ECC) — tooth decay among children younger than 6 years — is highly prevalent and consequential in the United States, despite being highly preventable. Forty-four percent of 5-year-olds have cavity experience (1). Early disease predicts lifelong cavities (2) because the process that results in cavities, once established, tends to be stable and chronic. ECC manifests frequently as pain and infection and disproportionately affects low-income children (3), leading to avoidable expenditures by Medicaid and the Children's Health Insurance Program (4). The problem facing policy makers is selecting interventions that have greatest potential for reducing both disease and costs.

System dynamics is "a methodology for better anticipating the likely effects of interventions in dynamically complex situations" (5). It maps the causes of a persistent problem and uses computer simulation to compare alternative policies and intervention strategies that might alleviate the problem. System dynamics has been applied to health care delivery and population health for chronic conditions affecting oral health and to various public health initiatives. Although it does not predict the future, system dynamics allows policy makers and program managers to consider resolution of complex, multilayered, interactive issues in an organized way.

The objective of this study was to formulate a system dynamics model to assess and compare ECC interventions for benefits and costs among young children in Colorado. Its framework can be applied to other locales.

## Methods

Our basic model structure, developed by a work group of pediatric medical, dental, and public health experts, separates children by age (0-6, 7-24, and 25-72 mo) and risk of developing ECC (low, moderate, high), using household income as a surrogate for risk. Risk is considered a key element in determining allocation of public health and dental care resources (6,7). Caries experience may vary over time with changes in socioeconomic status (8) and preventive interventions.

### Stages of ECC

**Figure 1.**
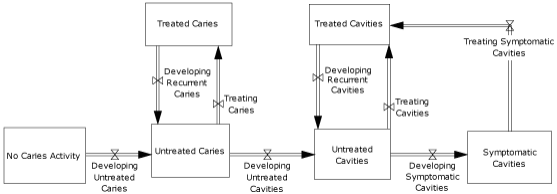
Stages of early childhood caries development among children aged 0 to 5 years. Symbols with 2 triangles touching at their vertices represent "valves," indicating that various factors control the rates of flow. These factors include both biological variables such as normal rates of caries development and effects of interventions such as fluoride varnish in slowing those rates of progression.

Caries is the disease process that causes cavities (Figure 1). The natural history of ECC begins with a newly erupted tooth that is not yet colonized by cariogenic bacteria. It progresses through the establishment of a biofilm that may be visible as plaque, followed by demineralization of the enamel resulting in a precavity lesion, or "white spot." With continued progression, the enamel breaks down, resulting in a cavity. In our modeling, all phases of this dynamic chain from initial biofilm formation to cavity are termed "caries," and the physical breakdown of the tooth structure (cavitation) is termed "cavities." A cavity becomes symptomatic when it progresses to affect dentin and pulp. Various preventive and disease management interventions may affect this progression at any point along the way. At each point in the simulation, children move in at least 2 directions as they age and as ECC stages progress. With intervention, children can move in an additional direction if the intervention is sufficient to change risk status.

We set up the model to continuously reflect the initial distribution of children among age, risk, and illness groups in the absence of any interventions. Interventions change rates of flow from one status to another and, over time, yield different patterns of ECC prevalence. The model calculates a number of variables including overall fraction of children with cavities and cumulative costs of restorative care so that program costs, cost savings for restorative care, and net costs can be attributed to different interventions and combinations of interventions.

### Data sources

We used multiple data sources to quantify the model and the effects of simulated interventions. To determine ECC prevalence by age and income and confirm the previously reported relationship between oral health and income, we obtained state-specific information from the Colorado Child Health Survey (9) (Rickey Tolliver, State of Colorado, written communication, November 5, 2009). We used a positive response to a question querying parents about children's prior dental pain, cavities, broken or missing fillings, or teeth pulled because of cavities as a surrogate for a finding of ECC on clinical examination. The National Health and Nutrition Examination Survey (NHANES) stratifies pediatric cavity experience by income levels of less than 100%, 100% to 199%, and 200% or more of the federal poverty level (FPL) (10), but Colorado data supported using income breaks of up to 200% of the FPL (18.6% ECC prevalence) for "high risk," 201% to 300% of the FPL (15.0% ECC prevalence) for "moderate risk," and 300% or more of the FPL (8.4% ECC prevalence) for "low risk." We categorized children for whom income data were not available (6.0% of respondents) as high-risk because they had similar ECC experience. Of the 431,070 children in Colorado's population of 0- to 5-year-olds, 31.4% were classified as high-risk, 17.8% were moderate-risk, and 50.8% were low-risk; 8.2% were aged birth to 6 months, 24.7% were 7 to 24 months, and 67.1% were 25 to 72 months. To quantify the fraction of children with untreated and treated cavities at baseline and to compensate for lack of parental knowledge about their children's nonsymptomatic cavity status, we adjusted Colorado findings to reflect treated and untreated cavity prevalence reported for the 1999-2002 NHANES survey after adjusting for Colorado's income distribution. A prior secondary analysis of these NHANES data (11) was used to calculate the selected Colorado income bands.

We derived the fraction of children with symptomatic cavities from the Government Accountability Office, which conducted a secondary analysis of NHANES data to determine the number of children considered to be "in urgent need of treatment" (12). To estimate precavity caries fractions, we backward-extrapolated the rate of change in cavities between age groups (between the youngest and middle age groups from the Colorado survey and between middle and older age groups from NHANES). Results were consistent with a study that measured both precavity lesions and cavities among children (13). We used the model to more finely calibrate rates of children moving between stages of tooth decay and finally adjusted the model by using data on rates of childhood dental fillings from the Medical Panel Expenditure Survey (MEPS) (14). We revised recurrence rates (new cavities among children with prior repair) upward to maintain the model's ability to reflect initial prevalence patterns. The model produces restorative visit rates that fall within the range suggested by MEPS data.

In addition to the prevalence of ECC, the model seeks to anticipate the extent of primary-tooth cavity experience, measured in average numbers of decayed and filled teeth. Using NHANES data (10), we adjusted for differences between national and Colorado's income distribution and rates of cavity experience for 6- to 24-month-olds and scaled downward from the rates for children younger than 6 years.

To calculate cost savings attributable to avoided restorative care from various interventions, we obtained data for care delivered in both dental offices and ambulatory or hospital sites under general anesthesia. For office-based care, MEPS reported average restorative dental costs of $216 in 2004 for all children, adjusted for inflation at 5% per annum to $276 in 2009 dollars. We attributed an average cost of $7,204 based on Medicaid data adjusted to reflect market-level charges for the 12% of children younger than 6 years requiring care under anesthesia reported by Colorado Medicaid (M. Sajovitz, Colorado Department of Health Care Policy and Financing, written communication, September 17, 2010) and the National Survey of Ambulatory Surgery (15) (M. Hall, Centers for Disease Control and Prevention [CDC] National Center for Health Statistics, written communication, August 4, 2010).

### Interventions analyzed

Among interventions of interest are those that delay transmission of cariogenic bacteria to children. To provide baseline values, the model was quantified with fractions of children by age and risk with detectable levels of cariogenic bacteria, specifically *Streptococcus mutans.* On the basis of studies of *S. mutans* prevalence among mixed-income (16) and low-income groups (17) and a putative "window of infection" of 19 to 31 months (18), we spread acquisition of *S. mutans* across the model's age groups. Simulations with the model were run over 10 years to determine changes in fractions of children younger than 6 years with cavities, untreated cavities, and symptomatic cavities; numbers of decayed and filled teeth; and costs of restorative care. We weighted results against estimated program costs to estimate costs, benefits, and net savings for various interventions. Interventions were applied to entire populations or to age or risk subgroups.

Interventions considered in the analysis included 1) educational programs that reduce consumption of sugary drinks, nocturnal bottle use, and other harmful behaviors; 2) efforts to reduce *S. mutans* transmission from parents and other caregivers to children using xylitol gum, chlorhexidine, or behavioral interventions; and 3) use of xylitol products directly with older children; 4) aggressive screening for and treatment of caries activity to reduce progression to cavities; 5) expanded use of fluoride varnish; 6) focused preventive care and education for children who already have cavities to reduce recurrence; 7) expansion of community water fluoridation to the entire population; and 8) motivational interviewing with strong educational and behavioral components. Motivational interviewing is a brief interactive approach to counseling and educating parents that focuses on skills that move patients to action. Interventions were clustered into 6 categories: fluoride exposure, transmission reduction, xylitol administration, clinical treatment, motivational interviewing, and combinations of these.

## Results

The Table reports projected disease levels in terms of the percentage of children with cavities, percentage of children with untreated cavities, and total numbers of decayed and filled teeth for the population. The Table also reports cumulative costs of restorative care, savings in restorative care compared to no intervention, and cumulative program costs. For example, simulation 1.1, community water fluoridation for all, projects a 6.6% relative reduction in cavity prevalence among Colorado children younger than 6 years (18.2% to 17.0%), a 0.8% relative reduction in their untreated cavities (71.4% to 70.8%), and 16,881 fewer affected teeth (265,923 to 249,042), at a cumulative 10-year cost reduction of $14 million ($208 million to $194 million) after expending $6 million on the intervention, for an $8 million net savings.

### Fluoride interventions

Simulation 1.1 assumes that community water fluoridation is expanded to 24.6% of Colorado's population not currently served; that fluoridation reduces cavity prevalence by 25.4%, half the average reduction reported by CDC (19) to compensate for other sources of pediatric fluoride exposure (William Maas, Pew Center on the States, written communication, September 28, 2010); and that the cost of fluoridation is $0.50 per person for the expanded population, including adults (20). The next 3 simulations consider variants of fluoride varnish application: to all children older than 6 months twice annually (1.2), to high-risk children older than 6 months 3 times annually (1.3), and to all children older than 24 months twice annually (1.4). All assume that fluoride varnish reduces decay of primary teeth by one-third (21,22) at a cost of $16 per child per application (William Maas, Pew Center on the States, written communication, September 28, 2010).

### Transmission interventions

Interventions aimed at reducing transmission of cariogenic oral bacteria to children, simulations 2.1 and 2.2, are modeled on assumptions of 88% *S. mutans* colonization reduction among 7- to 24-month-olds and 64% reduction among 25- to 72-month-olds when mothers are provided with education and treated to suppress their oral bacteria (23,24). Associated caries reductions of 73% are anticipated (25,26), at a cost of $100 per mother.

### Xylitol interventions with children

The effect of treating children with xylitol is reported only for children aged 24 months or older (27). Because research on xylitol interventions has reported both low-impact (44%) and high-impact (73%) reductions in cavity experience (28), simulations are modeled separately for each of these levels. Simulations 3.1 and 3.2 address all children aged 24 months or older, and simulations 3.3 and 3.4 address only those of this age group who are at high risk. Simulation 3.5 posits xylitol intervention beginning at age 7 months for children of all risk levels. The assumed cost of xylitol interventions is $100 per child.

### Secondary prevention

Simulations 4.1 and 4.2 assume white-spot lesions are identified and treated before they become cavities for children older than 6 months. Simulation 4.1 assumes a fraction of untreated caries that is treated per month equal to the model's rates for treating cavities, and 4.2 assumes a more aggressive program of screening and treatment. In simulations 4.3 and 4.4, follow-up care is intensified for children who have had prior restorative care to limit recurrence by 50% or 75%. The 2009-adjusted cost for caries management of $242 is the median between MEPS-reported costs for preventive and restorative care (14).

### Motivational interviewing

Motivational interviewing, with appropriate follow-up, can reduce cavity prevalence by 63% (29), at an estimated per-child cost of $100. Simulations 5.1 and 5.2 consider motivational interviewing for all children and for high-risk children.

### Combined interventions

Simulation 6.1 combines fluoride varnish for all children older than 6 months (1.2) with clinical treatment of active caries (4.1). Simulation 6.2 adds clinical care of cavities to reduce recurrence (4.3). Simulation 6.3 adds motivational interviewing (5.1). Combining interventions can have cumulative and complementary effects. Combining several interventions can produce a smaller fraction of children with cavities than can any of the single interventions.

## Discussion

This study, for the first time, synthesizes information from a wide variety of sources to formulate a model that compares ECC interventions for their effect on caries and their associated costs over time.

This study of Colorado's young children provides evidence that may be immediately applicable to policy making within and beyond the state. Expansion of community water fluoridation (1.1) was found to be cost-saving even in an environment where most of the population receives fluoridated water, and additional savings are projected from reduced treatment and retreatment costs for children older than 6 years who are not considered in the model. Fluoride varnish application for all children older than 6 months provides greater cavity reduction than does water fluoridation or other fluoride varnish interventions and saves more than half the program's cost in reduced restorative care but is the most expensive of the fluoride interventions. Starting fluoride varnish early (age >6 mo) results in greater disease reductions than starting later (age >24 mo) and pays back a larger percentage of program costs in reduced restorative costs (55% vs 32%). Targeting the highest-risk children has a similar effect on prevalence as fluoride varnish for all children older than 24 months but at a lower program cost. Thus, when funding is limited, priority should be considered for children at highest risk. Similarly, motivational interviewing yields a greater benefit per dollar expended when targeting high-risk children.

**Figure 2.**
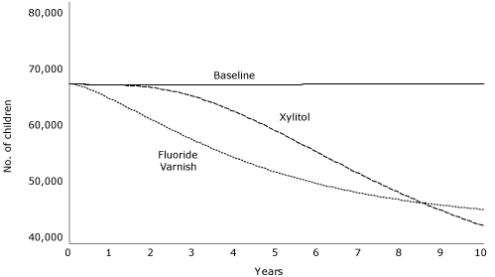
Number of Colorado children aged 2 to 5 years projected to experience cavities during a 10-year period, given 1 of 2 interventions in a simulation model, compared to baseline. The 2 interventions were xylitol treatment of mothers to limit cariogenic bacterial transmission and fluoride varnish application to all children aged 6 months or older.

Effects of xylitol interventions with children are limited by their late application. As with fluoride varnish, reductions in restorative costs pay back a substantial portion of the program costs. Xylitol for only high-risk mothers yields a smaller overall effect than for all mothers but larger proportional reductions in restorative costs. Applying this intervention to the high-risk group was projected to achieve a net savings of $3 million. Comparing fluoride varnish for all children with xylitol for all mothers (Figure 2) reveals that over time these 2 interventions play out differently and counterintuitively. Fluoride varnish results in more immediate cavity reductions among children older than 24 months, but reducing transmission by treating mothers results in greater long-term cavity reductions for this age group. A combined xylitol intervention for both mothers and children may be useful.

**Table:** 

Intervention	Beginning Value	End of Year				

		2	4	6	8	10
**Baseline**	66,368	66,191	66,240	66,307	66,353	66,381
**Fluoride varnish**	66,368	60,448	54,168	49,833	47,167	45,597
**Xylitol for moms**	66,368	65,761	61,799	55,108	48,255	42,865

Secondary prevention is effective in reducing the fractions of children with cavities and resulting restorative costs. There is no additional programmatic cost assumed in these simulations, although such a cost might be estimated. Preventive care aimed at reducing recurrence does not, by definition, affect the fraction with cavities but does reduce the fraction of children with untreated cavities. The effect of secondary prevention is also evident in the reduced cost for restorative care that would otherwise be required for children with recurrent cavities.

Taken together, findings of simulations that project the disease reduction and cost effects of various ECC interventions suggest that 1) interventions targeting the youngest children take 2 to 4 years longer to affect the entire population of preschool-age children but lead to greater reductions in ECC, 2) interventions targeting the highest-risk children provide the highest return on investment, 3) combined interventions that target ECC at several stages of its natural history can have the most profound effect. Primary prevention provides the greatest leverage, but limiting disease progression by screening for and treating caries before cavities form is also productive.

Although the model's framework is generalizable to any population of young children, findings will depend on the quality of the data inputs available, the geographic area considered, and the underlying rates of ECC in the target population. Needed to improve the model's validity are more reliable and current data sources. Applying the framework more widely will allow cross-state and cross-population comparisons that can be assessed for consistency or sensitivity to changes in underlying inputs.

The strengths of this model are its flexibility, capacity for long-term projections, ability to empirically derive findings that may not be anticipated, and value in comparing alternative interventions within complex systems. Weaknesses relate primarily to the quality of data used because proxies, estimates, expert opinions, and extrapolations were employed when data were not available. As with studies of other chronic disease interventions, system dynamics is valuable for policy makers who seek the most favorable outcomes and cost efficiencies in reducing population disease burden. We conclude from this work that ECC burden among children in Colorado can be markedly reduced through a combination of interventions designed to prevent and suppress the caries process that too commonly results in dental cavities.

## Figures and Tables

**Table.** Results of a Simulation Model for Designing Effective Interventions in Early Childhood Caries, Colorado

**Simulation**	Children With Cavities, %	Children Whose Cavities Are Untreated, %	Decayed and Filled Teeth, n	10-Year Cumulative Cost of Restorative Care, $ (Millions)	10-Year Savings of Restorative Care Compared to Baseline, $ (Millions)	10-Year Cumulative Program Cost, $ (Millions)
Baseline (no intervention)	18.2	71.4	265,923	208	0	0
**Fluoride interventions**						
1.1: Community water fluoridation for all	17.0	70.8	249,042	194	14	6
1.2: Fluoride varnish for children >6 mo	12.4	67.3	182,196	143	65	118
1.3: Fluoride varnish for high-risk children >6 mo	14.7	68.7	214,281	174	34	56
1.4: Fluoride varnish for all children >24 mo	16.0	67.8	233,823	181	27	85
**Transmission interventions in mothers**						
2.1: Xylitol for all mothers	10.8	68.2	160,609	152	56	79
2.2: Xylitol for mothers of high-risk children	15.0	69.3	201,765	180	28	25
**Xyiltol interventions in children**						
3.1: Xylitol for all children >24 mo, low-impact^a^	15.2	66.2	222,741	172	36	93
3.2: Xylitol for all children >24 mo, high-impact^b^	13.3	60.3	193,797	148	60	93
3.3: Xylitol for high-risk children >24 mo, low-impact^a^	16.9	68.4	238,711	189	19	29
3.4: Xylitol for high-risk children >24 mo, high-impact^b^	16.0	65.5	220,179	176	32	29
3.5: Xylitol for all children >6 mo, high-impact^b^	5.6	55.3	82,187	77	131	107
**Clinical treatment: caries treatment and prevention of recurrence**						
4.1: Caries treatment, children >6 mo, low treatment intensity	14.2	71.4	209,833	177	31	33
4.2: Caries treatment, children >6 mo, high treatment intensity	12.8	70.3	189,550	168	40	49
4.3: Prevention of recurrence, 50% reduction	18.2	62.7	265,923	186	22	0
4.4: Prevention of recurrence, 75% reduction	18.2	55.3	265,923	169	39	0
**Motivational interviewing^c^**						
5.1: Motivational interviewing for all families	6.5	59.4	95,434	86	122	111
5.2: Motivational interviewing for high-risk families only	12.9	66.0	159,020	144	64	35
**Combination interventions**						
6.1: Combination of 1.2 and 4.1	9.1	66.4	136,005	121	87	147
6.2: Combination of 1.2, 4.1, and 4.3	9.1	57.4	136,005	108	100	147
6.3: Combination of 1.2, 4.1, 4.3, and 5.1	3.8	42.4	56,158	59	149	245

a Low-impact assumes that xylitol interventions reduce caries at the low end (by 44%) of the reported range of impact (29).

b High-impact assumes that xylitol interventions reduce caries at the high end (by 73%) of the reported range of impact (29).

c Defined as a brief interactive approach to counseling and educating parents that focuses on skills that move patients to action.
